# The Effect of Dose and Timing of Dose on the Association between Airborne Particles and Survival

**DOI:** 10.1289/ehp.9955

**Published:** 2007-10-05

**Authors:** Joel Schwartz, Brent Coull, Francine Laden, Louise Ryan

**Affiliations:** 1 Department of Environmental Health and; 2 Department of Epidemiology, Harvard School of Public Health, Boston, Massachusetts, USA; 3 Channing Laboratory, Brigham and Women’s Hospital, Harvard Medical School, Boston, Massachusetts, USA; 4 Department of Biostatistics, Harvard School of Public Health, Boston, Massachusetts, USA

**Keywords:** air pollution, dose response, model averaging, particles, PM_2.5_, spline, survival, threshold, uncertainty

## Abstract

**Background:**

Understanding the shape of the concentration–response curve for particles is important for public health, and lack of such understanding was recently cited by U.S. Environmental Protection Agency (EPA) as a reason for not tightening the standards. Similarly, the delay between changes in exposure and changes in health is also important in public health decision making. We addressed these issues using an extended follow-up of the Harvard Six Cities Study.

**Methods:**

Cox proportional hazards models were fit controlling for smoking, body mass index, and other covariates. Two approaches were used. First, we used penalized splines, which fit a flexible functional form to the concentration response to examine its shape, and chose the degrees of freedom for the curve based on Akaike’s information criterion. Because the uncertainties around the resultant curve do not reflect the uncertainty in model choice, we also used model averaging as an alternative approach, where multiple models are fit explicitly and averaged, weighted by their probability of being correct given the data. We examined the lag relationship by model averaging across a range of unconstrained distributed lag models.

**Results:**

We found that the concentration–response curve is linear, clearly continuing below the current U.S. standard of 15 μg/m^3^, and that the effects of changes in exposure on mortality are seen within two years.

**Conclusions:**

Reduction in particle concentrations below U.S. EPA standards would increase life expectancy.

Epidemiology has traditionally dealt with identification of risk factors. However, for many such factors, including those in environmental and nutritional epidemiology, the risks occur within the common range of exposure. Hence for policy makers, identification of the shape of the exposure–response curve, and particularly whether there is a threshold dose, can be a key issue in decision making. For example, a large body of epidemiologic evidence has indicated that exposure to airborne particles from fossil fuel combustion is associated with early death (Katsouyanni et al. 1997, 2001; Ostro et al. 1999; Pope et al. 1999a; Samet et al. 2000; Schwartz 1994; Schwartz and Dockery 1992; Schwartz and Marcus 1990; Schwartz and Neas 2000). Most of this work has associated short-term changes in particle concentrations with short-term changes in daily deaths. However, two large cohort studies in the United States (Dockery et al. 1993; Pope et al. 2002) and one in Europe (Hoek et al. 2002) have demonstrated that, controlling for the standard risk factors, survival is shorter in more polluted towns.

More recently, other research has identified potential mechanisms for the association with shorter survival, such as changes in autonomic function (Creason et al. 2001; Gold et al. 2000; Pope et al. 1999b), perhaps leading to increased risk of arrhythmias (Peters et al. 2000), changes in inflammation and thrombotic factors (O’Neill et al. 2006; Peters et al. 1997, 2001b; Schwartz 2001; Zeka et al. 2006), potentially increasing the risk of myocardial infarctions (D’Ippoliti et al. 2003; Le Tertre et al. 2002; Peters et al. 2001a; Schwartz and Morris 1995), impaired endothelial function (Künzli et al. 2005; O’Neill et al. 2005), and exacerbation of respiratory illnesses (Zanobetti et al. 2000). Nevertheless, public officials, faced with the necessity of setting standards, have struggled to estimate the extent of life loss that could be avoided by reducing pollution at different levels. The U.S. Environmental Protection Agency (EPA) recently refused to tighten the annual average standard for particles (15 μg/m^3^), arguing that there is no convincing evidence for effects below that level (U.S. EPA 2006).

A second issue is that, because each person was assigned a single long-term exposure in the cohort studies, they provide little evidence as to when, after a change in exposure, we might expect to see changes in life expectancies: Are we looking at the effects of exposures over a lifetime, or effects of recent year’s exposure?

There are several approaches available to address these concerns. Concentration–response modeling often either assumes a parametric form for the relationship *a priori*, or chooses the best-fitting model from a set of parametric forms. A common parametric form is the piecewise constant model, such as dummy variables for quintiles of the exposure. The disadvantage of this approach lies in the relatively implausible assumption of a step function dose–response curve. Alternatively, some have attempted to estimate the relation nonparametrically, using smoothing splines or variants (Schwartz et al. 2002). Often piecewise polynomials are fit (Samoli et al. 2003). A common approach starts with more pieces than one expects to be necessary, and constrains the changes in slope among adjacent segments to be not too large, as in smoothing or penalized splines (Eilers and Marx 1996). The choice of constraint is made using some data-driven goodness-of-fit criteria. In the end, this strategy amounts to examining a range of alternatives, and choosing the best-fitting model, based on some goodness-of-fit criteria.

Similarly, for identifying the biologically relevant exposure lags, distributed lag models (Schwartz 2000) allow one to examine the issue of latency between exposure and response, as well as cumulative effects (Zanobetti et al. 2002). Incorporating multiple lags of an exposure in a model can lead to instability when the lagged exposures are correlated, and typically constraints are used to stabilize the results.

Unfortunately, in either case, alternatives that fit almost as well might have substantially different shapes. Although standard methods report uncertainties in parameters or curves, given the model that has been chosen, they do not incorporate uncertainties about the choice of model.

One approach that recognizes the inherent uncertainty in relating response and latency to exposure is Bayesian model averaging (BMA). This approach entails fitting a range of relationships, chosen to represent a reasonable space of possible alternatives. Rather than reporting the best-fitting alternative, one takes as the final estimate a weighted average of the model-specific estimates, weighted by the probability that a particular model is correct given the data. In this sense, the results are still data driven. This weight also incorporates any prior probabilities placed on the individual models. The resulting estimated uncertainty associated with the final estimate incorporates uncertainty associated with parameter estimates from each candidate model as well as model uncertainty. Thus, the approach recognizes, and accounts for, the fact that we do not know the model form with 100% certainty. Hoeting et al. (1999) provide a good introduction to this approach.

In addition, by allowing us to include in the models considered the option for a threshold at various concentrations, this approach allows us to say that we have explicitly examined those scenarios. If the final results do not look much like a threshold, we can say that the data provide considerably less support for those models than for alternatives. The same argument can be made for superlinear relations. This provides an intuitively appealing interpretation to the resulting concentration–response curve. We can argue that we gave everyone’s favorite relation a chance, and this is the result.

Dockery et al. (1993) examined the effects of long-term pollution exposure on survival of adults participating in the Harvard Six Cities Study followed for 14–16 years during the 1970s and 1980s. Exposure to particulate matter < 2.5 μm in aerodynamic diameter (PM_2.5_) was defined by the city-specific average during follow-up, ignoring the year-to-year fluctuations in those levels. The estimated mortality rate ratio was 1.13 [95% confedence interval (CI), 1.04–1.73] for a 10-μg/m^3^ increase in city-specific PM_2.5_ concentrations. Laden et al. (2006) recently extended the follow-up of the cohort until 1998, and confirmed that the association persisted in the second follow-up period. We have used the same data, but by using yearly variations in PM_2.5_ as a time-dependent covariate, we here examine the dose and lag relationship between exposure and the risk of mortality, using penalized splines and model-averaging approaches.

## Materials and Methods

The study population has been described previously (Dockery et al. 1993). Random samples of adults (*n* = 8,111) were recruited in 1974 in Watertown, Massachusetts; in 1975 in Kingston and Harriman, Tennessee, and from specific census tracts of St. Louis, Missouri; in 1976 from Steubenville, Ohio, and Portage, Wyocena, and Pardeeville, Wisconsin; and in 1977 in Topeka, Kansas. The study was reviewed by the Harvard School of Public Health Human Subjects Committee.

### Mortality follow-up

Vital status was determined by searching the National Death Index (NDI) for calendar years 1979 (when the NDI began) through 1998. Deaths from 1974 to 1979 were identified from next of kin and social security records (Dockery et al. 1993). Underlying cause of death was extracted from NDI records for deaths in 1979 and later. For deaths before 1979 a certified nosologist defined cause of death based on death certificate review (Dockery et al. 1993). Survival times were calculated as death date (or 31 December 1998 for surviving participants) minus enrollment date.

### Air pollution exposure estimates

Each participant’s exposure to air pollution each year was defined by that year’s concentrations of PM_2.5_ in that participant’s city. Concentrations of PM_2.5_ were measured at a centrally located air-monitoring station in each community starting in 1979 and ending in 1986–1988 depending on the city (Dockery et al. 1993). For the years after this monitoring (1986–1998) we estimated exposure to PM_2.5_ using monitoring data from the U.S. EPA. This methodology has been described in detail elsewhere (Laden et al. 2006). In brief, we created city-specific regressions predicting our measured PM_2.5_ using PM_10_ (particulate matter < 10 μm in aero-dynamic diameter) levels from U.S. EPA Air Quality System monitors located within a 80-km radius of the study city, season, and humidity corrected extinction coefficient data from nearby weather stations as predictors. These equations were used to predict PM_2.5_ in subsequent years. We calculated city-specific annual mean PM_2.5_ concentrations as the average of four quarterly means of daily data for each available year. For years before sampling, PM_2.5_ values were assumed equal to the earliest sampling year.

### Statistical analysis

We estimated adjusted mortality rate ratios for air pollution in Cox proportional-hazards regression models, treating air pollution as a time-varying covariate, and controlling for risk factors of mortality and potential confounders applied in the original analysis (Dockery et al. 1993). The 8,096 subjects with complete information were followed up annually. Each subject’s mortality experience in a year of follow-up was contrasted with the exposure in that year in that city. Subjects remaining alive at the end of 1 year of follow-up were entered for follow-up in the subsequent year. This continued until the subject died or was censored, in 1999. This approach provided > 162,000 person-years of follow-up to be analyzed. The analysis was stratified by sex and 1-year age groups, such that each sex–age group had its own baseline hazard. Each model included indicator variables for current or former smokers, number of pack-years of smoking (evaluated separately for current and former smokers), an indicator variable for less than a high school education, and a linear and quadratic function of baseline body mass index (weight in kilograms divided by height in meters squared).

### Specification of models: concentration response

#### Penalized spline model

Our first analysis fit a Cox proportional hazards model, as described above, but used a penalized spline to estimate the concentration response relation between annual PM_2.5_, as a time-varying covariate (the concentration in each year of follow-up), and mortality experience in the Six Cities cohort. We used Akaike’s information criterion (Akaike 1973) to decide how many degrees of freedom (up to a maximum of 18) to use in the spline to fit the concentration–response curve. This provided a plot of the resultant curve, and a test for the nonlinear portion (i.e., for the deviation from linearity in concentration response) using an approximate *F*-test (Rupert et al. 2003).

#### BMA model

Our first goal was to find a set of functions for the concentration–response curve that reasonably represented any such plausible relation. Because any differentiable function can be locally approximated as a straight line, a reasonable approximation to fitting any such curve is to specify a relationship that is piecewise linear, with the magnitude of the linear slope changing at a finite number of change points. This approach is also referred to as a linear spline model (Rupert et al. 2003). Moreover, this approach directly incorporates the potential for thresholds at a range of concentrations. The range of annual average PM_2.5_ concentrations in our data was from 8 to 40 μg/m^3^. We therefore considered piecewise linear functions with up to five slope changes. Those locations were at 10, 15, 20, 25, and 30 μg/m^3^. There were not enough data above 30 μg/m^3^ to justify a further division of the high exposure category. We considered a curve with no slope changes (i.e., linear), curves with one slope change (at any of the five possible locations), with two slope changes at any two of the above locations, and so on, all the way up to a model with all five change points entered into the model. This set of choices yields 2^5^, or 32, candidate models. This approach has the advantage of directly incorporating three biological phenomena that may play a role in particle health effects: a threshold model, which specifies that the curve has a slope at or near zero below one of the change points; superlinearity, which specifies the slope is higher below one of the lower change points; and a saturation model, which specifies that the curve has a slope at or near zero above a certain concentration. The aim of averaging over these candidate models was to search across a range of different combinations of slope changes that is wide enough to effectively approximate any plausible concentration–response curve.

Averaging results from models with different numbers of change points is straightforward. We used the fact that all the candidate models can be considered to be models with five change points, subject to constraints. In the linear model, the constraint is that the change in slope at each possible change point (e.g., 10, 15) is zero. The model with three change points constrains the slope change at two possibilities to be zero. Hence we can parameterize each model *k*, *k* = 1,…,32 using regression coefficients


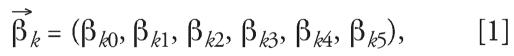


where β*_k_*_0_ is slope for PM values less than the first change point, and β*_kj_* is the change in slope at change point *j*, *j* = 1,…,5. The linear dose–response model is represented by (β_10_, 0, 0, 0, 0, 0). This model specifies all changes in slope are zero. We then average those slope changes with those estimated in the other 31 models, weighted according to how well each model fits the data. If the weights are high for models with, for example, the slope change at the third candidate change point being zero, then the estimated change in slope at that point in the model-averaged results will be low, and conversely.

#### Distributed lag

To examine the lagged association between exposure and risk of death, we considered models with only the same year’s exposure, with the same year’s exposure plus the previous year’s exposure, up to 5 years before the death. We also examined associations that started with the prior year’s exposure. This provided 11 possible alternatives as to which combinations of years were included. For each included year, we included a linear term for PM_2.5_ concentrations in that year.

#### Averaging models

The Bayesian framework specifies all model parameters and indicators reflecting whether a given candidate model is correct as random quantities. Inference is then based on the conditional distributions of these random variables given the data, also known as the posterior distributions. A natural weight for a given model in the model-averaging framework, then, is the posterior probability that a given model is correct given the data. In a fully Bayesian approach, this posterior probability for model *M_k_*, *k* = 1,...,32, is given by


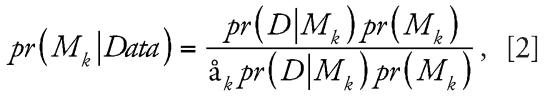


where


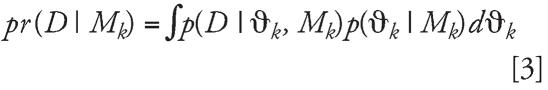


is the marginal distribution of the data given the model obtained by integrating over the distribution of the random parameters in that model, and *p*(*M_k_*) is the prior probability mass given the model. We assigned equal prior probability mass to each model, so that we did not *a priori* favor a particular candidate model. As a sensitivity analysis, we assigned all models with at least one slope change twice the prior probability of the linear no-threshold model, to see how much this influenced the results.

Unfortunately, calculation of the above integrals requires Monte Carlo simulation, which can be computationally prohibitive when the amount of data or the number of candidate models is large. With approximately 160,000 person-years of follow-up in the Six Cities Study and 32 candidate models, both of these limiting factors exist in our study. However, several authors (Buckland et al. 1997; Raftery 1995) have shown that model weights based on the Bayesian information criterion (BIC) (Volinsky and Raftery 2000) are an effective and computationally simple frequentist approximation to the posterior probability that a given model is correct. The BIC-based weights are


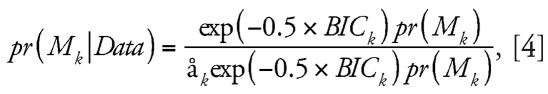


where *BIC_k_* is the value of the Bayesian information criterion for model *k*. Volinsky and Raftery (2000) showed that in the Cox proportional hazards model, replacing the number of observations in the standard formula for BIC with the number of events improves finite sample performance. We have used this approach to derive weights for our models.

We estimated standard errors for our results by dividing the sample into 50 groups and computing jacknife variance estimates for the parameters. This allowed us to incorporate covariances across models, and is a resampling alternative to the approximate formulas presented by Buckland et al. (1997).

## Results

**Figure 1:**
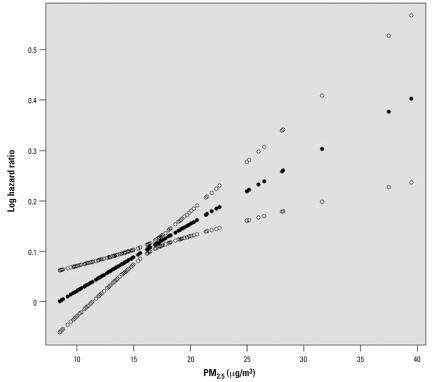
The estimated concentration–response relation between PM_2.5_ and the risk of death in the Six Cities Study, using a penalized spline with 18 knots. Also shown are the pointwise 95% CIs.

Table 1 shows descriptive statistics for the environmental variables in the study. Figure 1 shows the estimated concentration–response curve using the penalized spline model. It shows little deviation from nonlinearity, and the test for a nonlinear component of the curve was highly insignificant (*p* = 0.76).

**Figure 2:**
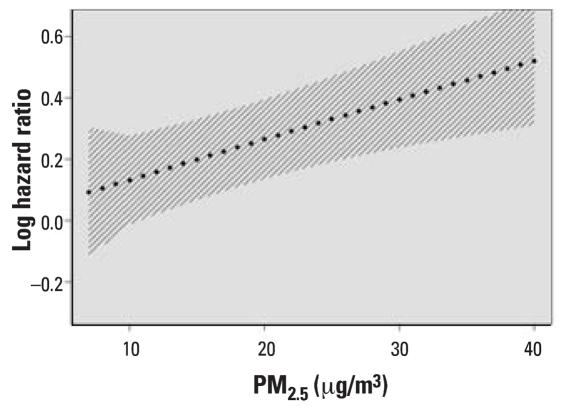
The estimated concentration–response relation between PM_2.5_ and the risk of death in the Six Cities Study, based on averaging the 32 possible models that were fit. Also shown are the point-wise 95% CIs around that curve, based on jacknife estimates.

**Figure 3:**
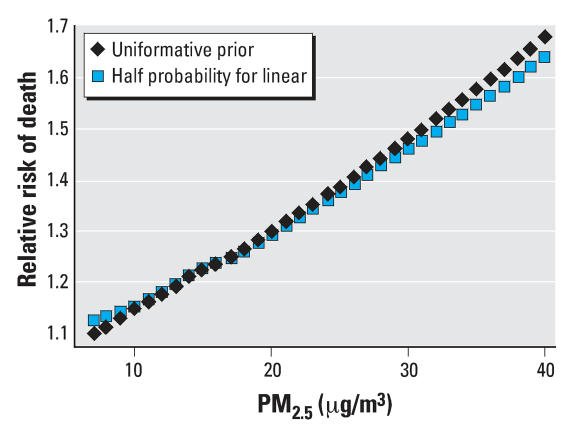
The estimated concentration–response relation between PM_2.5_ and the risk of death in the Six Cities Study, based on averaging the 32 possible models fit under the an uninformative prior, and under a prior giving a linear no-threshold model only half the probability of all other models. There is little difference in the two curves.

Table 2 shows the results of the BMA analysis. It lists the six (of 32) models for dose response that had posterior probabilities (based on the BIC approximation) of > 1%, as well as those posterior probabilities. The linear, no-threshold model had the great bulk of the probability, at 86%. The other models with nontrivial probability had a single slope change, at 10, 15, 20, 25, or 30 μg/m^3^ PM_2.5_ concentration. In all but one of these, the slope change was negative, indicating a somewhat lower slope at higher concentrations. The concentration–response curve, using the weighted average of all 32 models, is shown in Figure 2. It differs little from the curve generated by the penalized spline approach (Figure 1). Figure 3 shows the results of the sensitivity analysis where the linear no-threshold model was given half the prior probability of all other models. The results are indistinguishable except at the extreme ranges of the data, where there are few observations, and the prior would be expected to have more influence.

Because the concentration–response curve is indistinguishable from linear, the distributed lag modeling was done based on the linear model.

**Figure 4:**
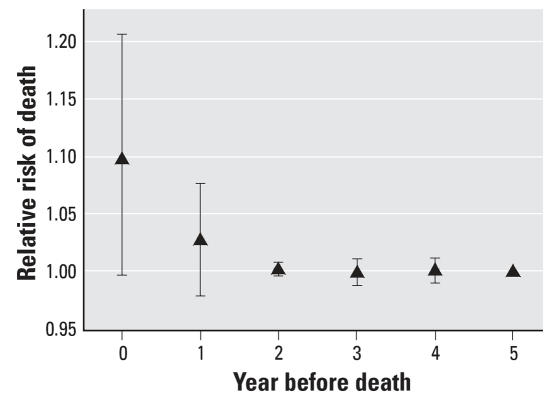
The model-averaged estimated effect of a 10-μg/m^3^ increase in PM_2.5_ on all-cause mortality at different lags (in years) between exposure and death. Each lag is estimated independently of the others. Also shown are the pointwise 95% CIs for each lag, based on jacknife estimates.

Table 3 shows the 11 candidate models for the distributed lag modeling, formed by considering different numbers of lags, and their posterior probabilities. Figure 4 shows the estimated relative risk (and 95% CI) for the effect of a 10-μg/m^3^ increase in PM_2.5_ in the year of death, the year preceding death, and so on, up to the 5 years preceding death. The increased risk of death associated with PM_2.5_ is essentially all manifested within 2 years of exposure.

**Figure 5:**
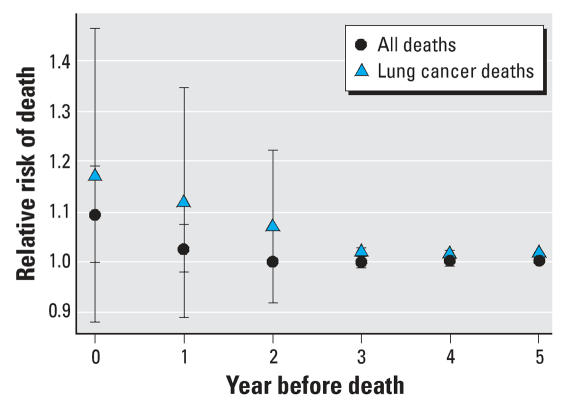
The model-averaged estimated effect of a 10-μg/m^3^ increase in PM_2.5_ on all-cause mortality and on lung cancer mortality. The estimated effect for lung cancer remains elevated up to 3 years preceding the death. Also shown are the pointwise 95% CIs for each lag, based on jacknife estimates.

Figure 5 compares the distribution of the effect by year of lag for all cause deaths (as in Figure 3) and deaths from lung cancer. The effect sizes for lung cancer are larger, and persist for a year longer than for all-cause deaths.

## Discussion

A key finding of this study is that there is little evidence for a threshold in the association between exposure to fine particles and the risk of death on follow-up, which continues well below the U.S. EPA standard of 15 μg/m^3^. Although similar results have been reported in time-series studies of the effects of daily particle levels on death the next day (Chuang et al. 2001; Daniels et al. 2000; Schwartz and Zanobetti 2000; Schwartz et al. 2002), this is the first detailed examination of the question in a cohort study examining annual exposures.

The apparent absence of a threshold has important implications. Air pollution standards that focus solely on reducing particle concentrations to an arbitrary standard will expose large populations to unnecessary risks in cities that meet the standard, but could reduce exposure further. Similarly, standards that focus on avoiding a few high pollution days are unlikely to be very effective in improving overall public health. A more reasonable goal is to try to reduce particle concentrations everywhere, at all times, to the extent feasible and affordable.

The finding that the deaths associated with exposure to fine particles occur primarily within a year or two of exposure also has important public health implications. It implies that policy changes that reduce air pollution can be expected to produce improvements in health almost immediately, with little delay between the expenditures that produce the improvement in air quality and the reductions in mortality that can be expected from those improvements. This has a major impact on cost–benefit analyses, which have been applied to air pollution standards.

That our study treats air pollution as a time-varying covariate has another advantage. In contrast to the original study (Dockery et al. 1993), exposure varies within a city in our analysis as well as between cities. Although previous cohort results have been shown to be robust to control for a large number of potential confounders (Krewski et al. 2005), one can never exclude confounding. In those studies, because exposure varied across cities, potential confounders also were those that varied across cities. In this study, exposure varies within city as well as among cities, reducing the potential for cross-sectional confounding. Our finding of essentially the same slope as previously reported suggests that any such confounding was small.

Finally, air pollution is not the only area where information about the shape of the exposure–response relation may be valuable for setting public health policy. The approach outlined here represents a feasible approach to addressing the issue, which explicitly addresses the possibility of thresholds.

Several studies have taken opportunistic advantage of sudden changes in pollution concentrations to address the same question we have. For example, Pope et al. (1992) examined mortality in Provo–Orem, Utah, during a 5-year period centered around a year when the steel mill that was the source of most of the particles in the valley was on strike. They showed that there was a 3% reduction in deaths in that year, compared with the previous and the following years. This finding indicates a rapid response of mortality to a change in annual average pollution. Clancy et al. (2002) examined the change in mortality after a sudden introduction of a ban on coal use for domestic heating in Dublin in 1990. These authors found a substantial drop in cardiopulmonary mortality after the ban. The drop appeared to have all happened in the first year; no further decline (or rebound) was evident in subsequent years.

We have considered two approaches to address the impact of model uncertainty on the shape of a dose–response curve, but there are certainly others. For instance, DiMatteo et al. (2001) and Dominici et al. (2002), among others, have considered free-knot spline approaches, which assume the number and placement of the knots in a linear spline model are random and simulate the posterior distributions of these quantities using a possible combinations of these factors using a Markov chain Monte Carlo (MCMC) approach. This approach can be somewhat tricky to implement because it employs a so-called reversible-jump MCMC approach to account for the change in model dimension that results from this non-nested set of models. In this article we focused on computationally simple approaches to this problem, because both the BIC approximation to formal Bayesian model averaging and penalized splines can be implemented in standard software packages.

One limitation of this study is the lack of personal monitoring, which it shares with all other published studies. One key advantage of the Six Cities Study is that subjects were recruited not from the cities at large, but from defined census tracts in compact neighborhoods within each city. The monitor was placed roughly in the middle of the neighborhood, which meant that most subjects lived within a few kilometers of the monitor. This results in much better exposure assignment than average. Indeed, the reanalysis of the American Cancer Society study (Jerrett et al. 2005), though based on spatial interpolation, had a similar spatial resolution. Another advantage of the Six Cities Study was that a random sample of the population was recruited in each neighborhood. Other cohort studies have relied on convenience samples, and therefore risk the possibility that their populations are distributed nonrandomly with respect to the monitors, possibly introducing bias as well as noise to the exposure assessment.

In conclusion, penalized spline smoothing and model averaging represent reasonable, feasible approaches to addressing questions of the shape of the exposure–response curve, and can provide valuable information to decision-makers. In this example, both approaches are consistent, and suggest that the association of particles with mortality has no threshold down to close to background levels.

## Figures and Tables

**Table 1:** Descriptive statistics for Six Cities Study follow-up.

Variable	Mean ± SD or proportion
Dead by end of follow-up	0.34
Lung cancer death	0.028
Smoker (at entry)	0.36
Former smoker (at entry)	0.24
Female	0.55
< High school education	0.28
Pack-years (in current smokers)	26 ± 20
Pack-years (in former smokers)	21 ± 22
Body mass index (at entry)	25.8
PM_2.5_ (μg/m^3^) (annual)	17.5 ± 6.8

**Table 2:** Six models (of the 32 models for dose response that were considered) with the highest posterior probability, and the posterior probability of each model, given the data.

	Knot location						
Model	0	10	15	20	25	30	Posterior probability
1	0.1211	0	0	0	0	0	0.860
2	−0.2575	0.3973	0	0	0	0	0.040
3	0.1286	0	−0.0105	0	0	0	0.016
4	0.1569	0	0	−0.0775	0	0	0.022
5	0.1493	0	0	0	−0.1038	0	0.024
6	0.1438	0	0	0	0	−0.1500	0.024

Values given are the coefficients for a 10-μg/m^3^ increase in PM_2.5_ for linear terms beginning at each change point.

**Table 3:** Eleven models for the distributed lag between exposure and death that were considered, and the posterior probability of each model, given the data.

Model	Lag 0	Lag 1	Lag 2	Lag 3	Lag 4	Lag 5	Posterior probability
1	0.1388	−0.0461	0.0825	−0.5528	0.4267	0.0499	0
2	0.1214	0	0	0	0	0	0.718
3	0.2147	−0.0939	0	0	0	0	0.018
4	0.2030	−0.1205	0.0376	0	0	0	0
5	0.2154	−0.0601	0.1992	−0.2214	0	0	0
6	0.1431	−0.0412	0.0792	−0.5366	0.4575	0	0
7	0	0.1074	0	0	0	0	0.255
8	0	0.0062	0.1011	0	0	0	0.007
9	0	0.0711	0.2566	−0.2139	0	0	0
10	0	0.0444	0.1121	−0.5472	0.4821	0	0.002
11	0	0.0352	0.1152	−0.5671	0.4428	0.0622	0

Values given are the coefficients for a 10-μg/m^3^ increase in PM_2.5_ for each lag between exposure and death.
